# Editorial: The role of vitamin D in reducing the risk of metabolic syndromes

**DOI:** 10.3389/fendo.2023.1293262

**Published:** 2023-10-02

**Authors:** Ziad H. Al-Oanzi, Abdel-Naser Elzouki

**Affiliations:** ^1^ Department of Clinical Laboratories Sciences, College of Applied Medical Sciences, Jouf University, Sakaka, Saudi Arabia; ^2^ Department of Medicine, Hamad General Hospital, Hamad Medical Corporation, Doha, Qatar; ^3^ Department of Medicine, College of Medicine, Qatar University, Doha, Qatar; ^4^ Department of Medicine, Weill Cornel Medical College, Doha, Qatar

**Keywords:** vitamin D, metabolic syndromes, obesity, diabetes, cardiovascular disease

Vitamin D (VitD) is important for several physiological and functional processes inside the human body, as shown by the presence of its receptors in all kinds of cells. VitD has an important role as a secondary hormone in several bodily tissues. Numerous studies have substantiated the link between insufficient levels of VitD and metabolic disruptions. Among these disturbances, the most notable mechanism involves the elevation of body fat resulting from heightened triglyceride levels, alongside increased cholesterol, blood glucose levels, blood pressure, and markers of inflammation (1–3). Consequently, such a condition predisposes the body to the progress of perilous chronic diseases, posing a significant threat to overall health. Laboratory studies, epidemiological research, and clinical investigations all provide evidence suggesting a potential association between VitD insufficiency and the onset and progression of obesity, diabetes mellitus (DM), cardiovascular disease (CVD), cancer, hepatic and renal disease and others (4). Scientific study endeavors to uncover preventative measures for chronic diseases by elucidating the function of VitD in mitigating the risk of metabolic abnormalities associated with these syndromes (**Figure 1**).

**Figure 1:**
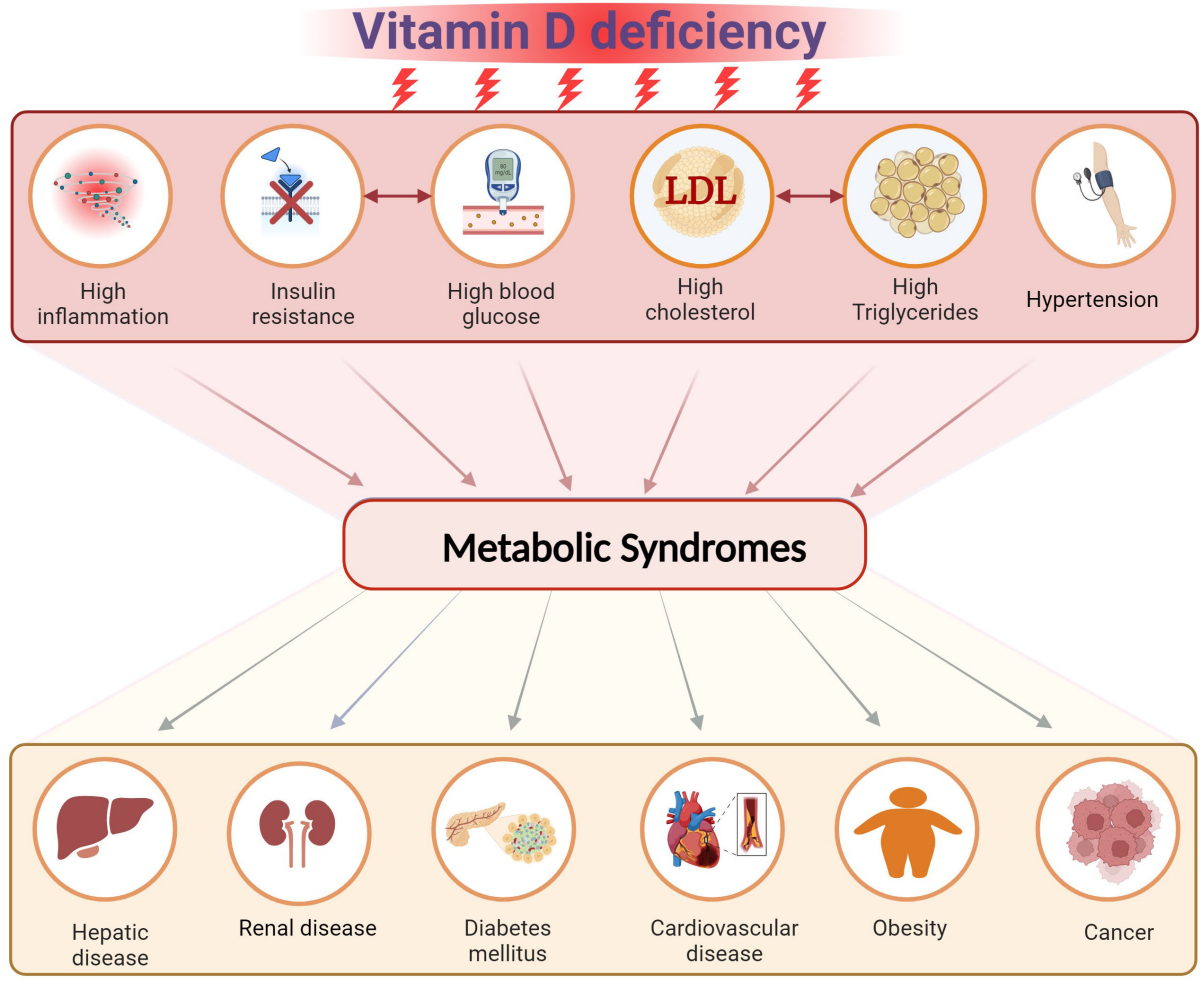
The effect of vitamin D deficiency on the risk of developing metabolic syndromes. Created with BioRender.com.

A discrepancy has been noted in the metabolism of VitD in individuals with a higher body mass index (BMI). Several investigations have demonstrated a consistent inverse relationship between BMI and the efficacy of VitD supplementation on bodily response (5, 6). This connection may provide a possible rationale for the discrepancies seen in the efficacy of VitD supplementation across persons with differing levels of obesity. The study done by AlAnouti et al. aimed to examine the association between blood 25-hydroxyvitamin D levels, body mass index (BMI), and body fat composition (BFC). The research was conducted on the Emirati people. The results indicate a significant relationship between BFC and VitD deficiency, suggesting that BFC may be a more accurate indicator than BMI. Further research is required to elucidate and substantiate the correlation between BFC and its superiority over BMI in terms of accuracy and benefits.

In a distinct investigation conducted by Du et al. research has shown that, VitD has a regulatory influence on hepatic fatty acids via its modulation of lipid uptake and beta-oxidation. The findings of this investigation indicate that non-alcoholic fatty liver disease (NAFLD) is associated with compromised hepatic functionality, resulting in dysregulation of blood lipid profiles and insulin sensitivity. The effectiveness of VitD in activating the peroxisome proliferator-activated receptor α (PPARα) signaling pathway was compared to that of a selective inhibitor through a comparative analysis. The findings demonstrated that the presence of the inhibitor abolished the anti-steatosis properties of VitD, suggesting that VitD may ameliorate hepatic steatosis via the PPARα signaling pathway. The present work is subject to many limitations, including the absence of quantification of proteins related to lipid synthesis and secretion. Furthermore, there is a lack of estimation about gene expression levels to validate the observed protein levels. However, more assessment via *in vivo* trials is required.

VitD is essential for healthy bone and lipid metabolism as well as for reducing cancer risk (7). The research conducted by Bao et al. identified a correlation between VitD insufficiency and aberrant bone and lipid metabolism. They also found that this deficit is related with an increased chance of developing multiple myeloma, a kind of cancer with a bad prognosis. The frequency of VitD insufficiency is notably elevated among people diagnosed with multiple myeloma (MM). In order to demonstrate this, a study effectively employed the ratio of serum VitD to carboxy-terminal telopeptide of type I collagen (β-CTX) to assess the diagnosis of myeloma. The study concluded that β-CTX levels play a significant role in the association between VitD deficiency and the clinical prognosis of persons make a diagnosis with newly diagnosed multiple myeloma (NDMM). Clinical data recommend that the ratio of VitD to β-CTX in patients with NDMM is an important predictor for identifying high-risk individuals with a poor prognosis. Furthermore, it provides evidence-connecting VitD with the onset and development of the condition.

The research conducted by Huang et al. demonstrated a correlation between the concentrations of 25-hydroxyvitamin D in blood serum and the occurrence of albuminuria in individuals with diabetic kidney disease. The study also incorporated various methodologies to investigate the potential mechanisms underlying the pathogenesis of this disease and its progression, employing bioinformatic analysis techniques. This study demonstrated a significant association between diabetic retinopathy (DR) and decreased glomerular filtration rate (eGFR) in individuals who developed microalbuminuria and subsequently progressed to macroalbuminuria. Moreover, it was observed that a more pronounced progression of albuminuria and reduced renal function at low serum 25-hydroxyvitamin D concentrations might indicate the involvement of VitD in additional mechanisms beyond the renin-angiotensin-aldosterone system (RAAS) pathway.

The research conducted by Herrera-Martinez et al. examined the efficacy of bariatric surgery and the administration of calcifediol and Gordian complex in the management of comorbidities associated with obesity. A primary purpose of this research was to comprehensively evaluate the alterations in VitD metabolites within the endocrine system, as well as relevant components of the inflammatory machinery and factors associated with inflammation. This paper is the first documentation establishing a connection between notable inflammatory constituents, endocrine VitD metabolites, and the amelioration of metabolic disorders in a clinical setting. Significantly, the findings of this study indicate that the expression pattern of inflammatory machinery components in peripheral blood mononuclear cells (PBMCs) can be altered in response to bariatric surgery and calcifediol treatment. It suggests that the aforementioned expression profile has the potential to function as a sensor and early indicator for the amelioration of obesity-related complications subsequent to bariatric surgery.

In conclusion, this Research Topic presents a diverse body of information that substantiates the prevailing issue in public health, namely the deficiency of VitD, which significantly affects the physiological mechanisms underlying chronic diseases. The principal consequences of vitamin D insufficiency in individuals with metabolic disorders are the development of low bone density, obesity, DM, and CVD.

## Author contributions

ZA-O: Writing – original draft, Writing – review & editing. ANE: Writing – review & editing

## References

[1] Al-Oanzi ZH Alenazy FO Alhassan HH Alruwaili Y Alessa AI Alfarm NB. The role of vitamin D in reducing the risk of metabolic disturbances that cause cardiovascular diseases. J Cardiovasc Dev Dis (2023) 10(5):209. doi: 10.3390/jcdd10050209 37233176 PMC10219128

[2] Melguizo-Rodríguez L Costela-Ruiz VJ García-Recio E De Luna-Bertos E Ruiz C Illescas-Montes R. Role of vitamin D in the metabolic syndrome. Nutrients (2021) 13(3):830. doi: 10.3390/nu13030830 33802330 PMC7999005

[3] Warren T McAllister R Morgan A Ra TS McGilligan V Ennis M. The interdependency and co-regulation of the vitamin D and cholesterol metabolism. Cells (2021) 10(8):2007. doi: 10.3390/cells10082007 34440777 PMC8392689

[4] D’souza S Udavant P Kadam J Khairnar S Ahire ED Sable R. “Role of vitamins in metabolic diseases.” Vitamins as nutraceuticals: recent advances and applications (2023). p. 205–33. doi. 10.1002/9781394175543.ch9

[5] Tobias DK Luttmann-Gibson H Mora S Danik J Bubes V Copeland T. Association of body weight with response to vitamin d supplementation and metabolism. JAMA Network Open (2023) 6(1):e2250681–e2250681. doi: 10.1001/jamanetworkopen.2022.50681 36648947 PMC9856931

[6] Serrano NC Rojas LZ Gamboa-Delgado EM Suárez DP Acosta IS Romero SL. Efficacy of vitamin D supplementation in reducing body mass index and lipid profile in healthy young adults in Colombia: a pilot randomised controlled clinical trial. J Nutr Sci (2023) 12:e29. doi: 10.1017/jns.2022.108 36843975 PMC9947753

[7] Ismail NH Mussa A Al-Khreisat MJ Mohamed Yusoff S Husin A Johan MF. The global prevalence of vitamin D deficiency and insufficiency in patients with multiple myeloma: A systematic review and meta-analysis. Nutrients (2023) 15(14):3227. doi: 10.3390/nu15143227 37513645 PMC10386623

